# Dogmatism manifests in lowered information search under uncertainty

**DOI:** 10.1073/pnas.2009641117

**Published:** 2020-11-19

**Authors:** Lion Schulz, Max Rollwage, Raymond J. Dolan, Stephen M. Fleming

**Affiliations:** ^a^Wellcome Centre for Human Neuroimaging, Institute of Neurology, University College London, London WC1N 3BG, United Kingdom;; ^b^Department of Experimental Psychology, University College London, London WC1H 0AP, United Kingdom;; ^c^Department of Computational Neuroscience, Max Planck Institute for Biological Cybernetics, 72076 Tübingen, Germany;; ^d^Max Planck University College London Centre for Computational Psychiatry and Ageing Research, Institute of Neurology, University College London, London WC1B 5EH, United Kingdom

**Keywords:** information search, dogmatism, politics, metacognition, computational modeling

## Abstract

When knowledge is scarce, it is adaptive to seek further information to resolve uncertainty and obtain a more accurate worldview. Biases in such information-seeking behavior can contribute to the maintenance of inaccurate views. Here, we investigate whether predispositions for uncertainty-guided information seeking relate to individual differences in dogmatism, a phenomenon linked to entrenched beliefs in political, scientific, and religious discourse. We addressed this question in a perceptual decision-making task, allowing us to rule out motivational factors and isolate the role of uncertainty. In two independent general population samples (*n* = 370 and *n* = 364), we show that more dogmatic participants are less likely to seek out new information to refine an initial perceptual decision, leading to a reduction in overall belief accuracy despite similar initial decision performance. Trial-by-trial modeling revealed that dogmatic participants placed less reliance on internal signals of uncertainty (confidence) to guide information search, rendering them less likely to seek additional information to update beliefs derived from weak or uncertain initial evidence. Together, our results highlight a cognitive mechanism that may contribute to the formation of dogmatic worldviews.

## Significance

Dogmatic individuals are reluctant to seek out new information to refine their views, often skewing political, scientific, and religious discourse in the process. The cognitive drivers of this reluctance are poorly understood. Here, we isolate an influence of uncertainty on information search using a low-level perceptual decision-making task. We show that people with dogmatic views are both less likely to seek information before committing to a decision and to use fluctuations in uncertainty to guide their search. Our results highlight a cognitive mechanism that may contribute to the formation of dogmatic worldviews.

A never-ending flow of informational choices is a defining feature of modernity (1). We are in charge of gathering information critical to our health (2), the survival of democracies (3), or the conservation of the planet (4). These decisions to seek information are in turn a crucial determinant of our beliefs. Cognitive science has studied information search extensively, providing us with a rich empirical and theoretical perspective on these choices (5–8). This research indicates that people prefer to seek information that confirms their beliefs and has positive valence, as when we read an additional news story about the victory of a favored political party. This type of motivated search is evident both in laboratory experiments (9–11) and in real-world data (12–14).

In contrast, normative frameworks propose that uncertainty, rather than valence, should determine where and when we should seek information (5, 7, 15). In the absence of external feedback, humans rely on internal confidence signals to guide such uncertainty-based information search. Bayes-optimal agents should seek out more information when they have lower confidence in a decision, because the likelihood of a mistake will then outweigh the cost of further information (16). Empirical data bear out such predictions (17, 18), showing that people are more likely to seek information when they express low confidence (i.e., higher uncertainty) in their decisions.

In everyday situations, both motivational influences and failures in uncertainty-guided information search can lead to biased or inaccurate beliefs, albeit via distinct mechanisms. For example, a person who does not believe in climate change is likely to show a preference for media that refutes its occurrence (19), reinforcing preexisting beliefs. Alternatively, people with doubts about the science of global warming (20) might fail to act on this uncertainty, and as a consequence not seek out further, potentially corrective evidence. In both of these cases, an unwillingness to seek out corrective information is one potential source of dogmatism, a worldview that involves a rigid maintenance of one’s beliefs (21–23) regardless of their accuracy (24). The scope of this worldview is wide-ranging and transcends specific issues and positions, affecting political (25), scientific (26), and religious debate (21, 26). Prior questionnaire-based research suggests a link between such a dogmatic style of thinking and a willingness to seek further information (21, 27). However, how motivation and uncertainty contribute to this phenomenon remains unknown.

Here, we address this question using a precise assay of uncertainty-guided information search in the context of a low-level perceptual decision-making task. Leveraging the computational precision afforded by this approach, we test (in both exploratory and replication samples) whether individual differences in a sensitivity to uncertainty explain a disposition to hold dogmatic beliefs. Our approach builds on previous research on the influence of confidence on information search (17, 28) and allows us to rule out possible motivational influences: Participants are unlikely to approach such a low-level task with vested interests or prior knowledge, and should not hold differing appraisals of the helpfulness of further information. Moreover, eliciting trial-by-trial estimates of confidence enabled us to infer how participants use uncertainty to guide their search.

## Results

We studied a sample of 370 US adults (study 1) and replicated all key findings in an independent second sample of 364 US participants (study 2). Both samples were recruited through Amazon Mechanical Turk and comprised a wide range of ages and educational backgrounds (see *Materials and Methods* and *SI Appendix* for details). Participants first completed an information-seeking task and then answered a number of questionnaires that allowed us to measure general belief rigidity and dogmatism, political beliefs, authoritarianism, and intolerance to opposing political attitudes (see *Materials and Methods* for details regarding the questionnaires). This methodology builds on our previous research as to how dogmatic individuals construct a sense of confidence (24).

### Measuring Dogmatism.

**Fig. 1.**
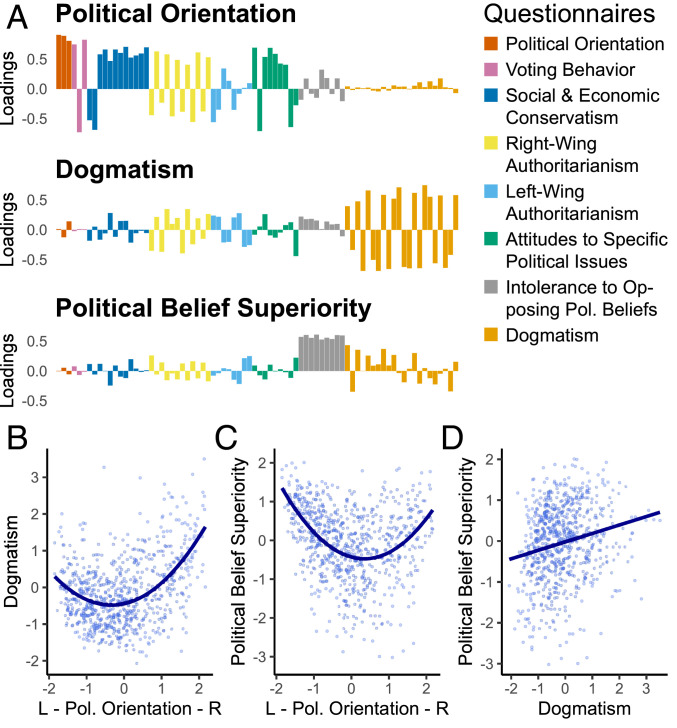
Liberal and conservative extremes of the political spectrum are associated with higher levels of dogmatism and belief superiority. (*A*) A factor analysis revealed a three-factor structure underlying responses to multiple questionnaires assessing political convictions, authoritarianism, and belief rigidity. The three factors identified 1) “political orientation” (liberal to conservative), 2) “dogmatism” representing a domain-general belief certainty, and 3) “political belief superiority” characterizing participants’ confidence in specific political convictions. Item loadings for each question are presented with individual questionnaires indicated by colors. (*B*–*D*) We examined the relationships between these constructs by investigating individual scores for each factor (combined data from studies 1 and 2 are plotted). (*B* and *C*) We observed that a combined linear-quadratic model provided the best fit to the relationship between both political orientation and dogmatism as well as between political orientation and political belief superiority. (*D*) The relationship between dogmatism and political belief superiority was best characterized by a linear relationship (see *SI Appendix* for further details about the factor analysis).

We derived a measure of dogmatism from a factor analysis applied to the questionnaire battery (Fig. 1). The breadth of the battery allowed us to quantitatively distinguish dogmatism from other, possibly related, constructs and study their interplay. The most parsimonious factor structure contained three factors, capturing 40% of questionnaire variance. A first factor represented individuals’ position on a left–right political spectrum, and a second factor described their domain-general dogmatism and rigidity of worldview (21). A third factor captured variance in beliefs as to the superiority of participants’ policy preferences (a factor related to but also theoretically independent of dogmatism) (22). Whereas this last factor is specific to political policy, dogmatism itself is a broader construct that describes the general way beliefs are held and acted upon (23, 29, 30). Dogmatism thereby transcends specific political preferences as evident, for example, in a link between dogmatism and religious fundamentalism (21).

We first explored interrelationships between individuals’ political orientation, dogmatism, and political belief superiority (*SI Appendix*, Table S2). We found both positive linear (study 1: *β*_linear_ = 0.16, *P* = 0.001; study 2: *β*_linear_ = 0.24., *P* < 10^−6^) and quadratic (study 1: *β*_quadratic_ = 0.35, *P* < 10^−13^; study 2: *β*_quadratic_ = 0.37, *P* < 10^−13^) relationships between political orientation and dogmatism across both samples. These findings indicate that individuals on both the far left and far right of the political spectrum show enhanced dogmatism, but interestingly this increase in dogmatism is more marked for those on the far right (see Fig. 1, *Materials and Methods*, and *SI Appendix*, Table S1 for further information). Conversely, a negative linear (study 1: *β*_linear_ = −0.33, *P* < 10^−10^; study 2: *β*_linear_ = −0.32, *P* < 10^−9^) and positive quadratic (study 1: *β*_quadratic_ = 0.43, *P* < 10^−20^; study 2: *β*_quadratic_ = 0.34, *P* < 10^−10^) relationship between political orientation and political belief superiority reveals that individuals on both the far left and far right show heightened beliefs in the superiority of their respective policy positions, but more so on the far left. Finally, we found a positive linear relationship between political belief superiority and dogmatism, indicating more dogmatic subjects also tended to be more confident in the superiority of their specific political convictions (study 1: *β* = 0.26, *P* < 10^−06^; study 2: *β* = 0.14, *P* = 0.006).

### Measuring Information Search.

**Fig. 2.**
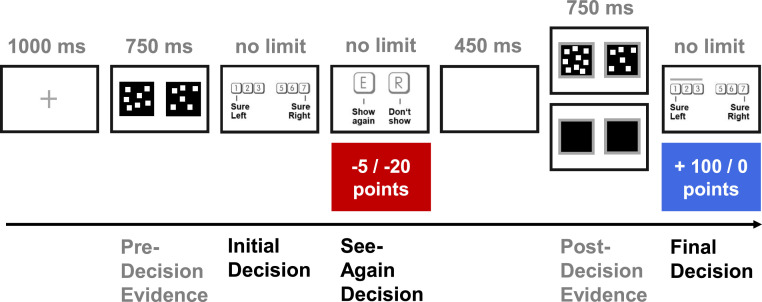
Example experimental trial. Participants first had to judge whether a left or right square contained more flickering dots. They then chose whether they wanted to see a stronger, more helpful, version of this stimulus again, costing them either 5 or 20 points. After seeing either this additional stimulus or blank boxes, they again made a judgment as to which box contained more dots. We compensated participants for the accuracy of this final decision alone (100 points for a correct judgment; 0 points for an incorrect judgment). Participants rated their confidence (on a six-point scale from “sure left” to “sure right”) at both the initial and the final decision. The difficulty of the initial decision was fixed through an individually predetermined difference in dot number that resulted in ∼71% accuracy. The post decision-evidence strength was yoked to this predetermined dot difference, to make the final decision easier (information on stimulus calibration is provided in *Materials and Methods* and *SI Appendix*).

We next tested our primary hypothesis of a link between dogmatism and uncertainty-guided information search. To probe this, we presented participants with a perceptual information-seeking task (Fig. 2; see *Materials and Methods* for details) where they received monetary reward for correctly judging which of two flickering boxes contained the greater number of dots. On each trial, participants were first presented with an initial pair of boxes, made an unincentivized judgment as to which box contained more dots and simultaneously reported their confidence in this judgment. Following this initial decision, participants were asked whether they wanted to see additional evidence so as to improve their decision accuracy. If participants decided to see this additional information, they were presented with another set of flickering dots. This additional information was always helpful (the correct option continued to have a greater number of dots) and was presented at a higher stimulus strength (greater dot difference). We imposed a variable cost for seeing this information through a deduction of points, allowing concurrent assessment of participants’ sensitivity to information cost. If participants decided not to see more information, they instead saw two empty black boxes for an identical duration and were not deducted any points. Finally, regardless of whether subjects decided to see additional information or not, they were asked to provide a final decision and confidence rating. To incentivize subjects to strive for the best possible overall accuracy, they were paid only for their performance on this final decision.

First, we validated that participants used the additional information adaptively (see *Materials and Methods* for details and *SI Appendix*, Fig. S2 for overview). Participants chose to see additional information more often after initial mistakes (study 1: *β* = −0.76, *P* < 10^−69^; study 2: *β* = −0.77, *P* = 10^−52^) and were more likely to make an accurate final decision after deciding to see additional information (study 1: *β* = 1.23, *P* < 10^−80^; study 2: *β* = 1.12, *P* < 10^−74^). Similarly, examining individual differences in information search revealed that participants who sought additional information more often also performed better in their final decisions (study 1: *β* = 0.68, *P* < 10^−50^; study 2: *β* = 0.71, *P* < 10^−56^) and received a higher payoff (study 1: *β* = 0.43, *P* < 10^−17^, study 2: *β* = 0.49, *P* < 10^−22^). Importantly participants were also sensitive to cost, seeking information less often when it was more expensive (study 1: *β* = −1.22, *P* < 10^−58^; study 2: *β* = −1.34, *P* < 10^−66^). Finally, participants sought out additional evidence less often when they were more confident in their initial decision (study 1: *β* = −2.02; *P* < 10^−126^; study 2: *β* = −1.93, *P* < 10^−130^), demonstrating that internal signals of uncertainty were used to guide information search.

### Information Search and Dogmatism.

**Fig. 3.**
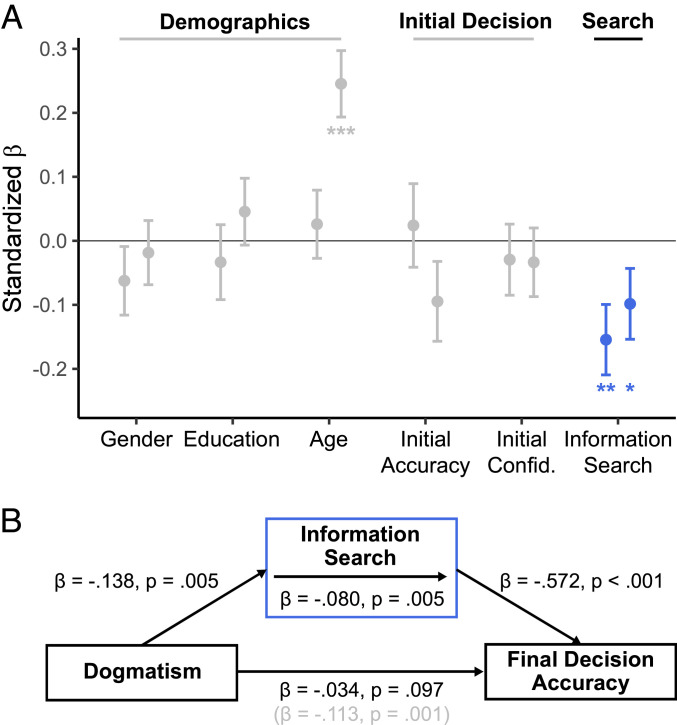
Dogmatism is characterized by a reduction in information search, leading to less veridical judgments. (*A*) Dogmatism was predicted by a reduced willingness to seek out more information before committing to a decision, controlling for several demographic and task variables. We present standardized *β* coefficients ± SE of predictors for study 1 (left markers, *n* = 370) and study 2 (right markers, *n* = 364). Effects in study 2 were tested one-tailed based on the directional hypothesis derived from study 1. *P < 0.05, **P < 0.01, and ***P < 0.001. A significant effect of age was found only in study 2, which we further discuss in *SI Appendix*. (*B*) A reduction in information search mediated less accurate overall final judgments in more dogmatic participants (mediation results for study 1 are presented in the figure; see main text for results from study 2).

We next asked whether more dogmatic participants differed in their propensity to seek out information. To that end, we sought to explain variance in dogmatism factor scores using behavioral measures derived from the information-seeking task. In line with our hypothesis, higher levels of dogmatism were associated with a reduced willingness to seek out information (study 1: *β* = −0.15, *P* = 0.005, *R*^2^ = 0.02; Fig. 3*A*). No significant relationships with initial decision accuracy (study 1: *β* = 0.02, *P* = 0.72) or overall confidence level (study 1: *β* = −0.03, *P* = 0.61) were found, and our analyses controlled for key demographic variables including age, gender, and education (see Fig. 3*A* and *Materials and Methods* for details). We replicated this lowered tendency for dogmatic subjects to seek out information in a second, independent sample in study 2 (*β* = −0.10, one-tailed *P* = 0.039, *R*^2^ = 0.01; Fig. 3), again in the absence of differences in initial decision accuracy (*β* = −0.09, *P* = 0.13) and confidence (*β* = −0.03, *P* = 0.53). To assess the overall strength of the relationship between dogmatism and information seeking, we conducted an internal meta-analysis of this effect by pooling data from both samples (31). This revealed a consistently negative effect across both samples (*β* = −0.12, *P* = 0.002, *R*^2^ = 0.012). Overall, our findings highlight that, in the absence of motivational factors, more dogmatic participants seek out less information before committing to a decision—even when this information would be helpful.

A key question arising from this finding is whether more dogmatic individuals’ final accuracy and payoff suffered because of their lowered information search, or whether they simply sought information more efficiently. Here, a mediation analysis (Fig. 3*B*; see *Materials and Methods* for details) showed that more dogmatic participants were in fact less accurate in their final decision (total effect: study 1, *β* = −0.11, *P* = 0.001; study 2, *β* = −0.09, one-tailed *P* = 0.01), and that this effect was fully mediated by a lowered willingness to seek information (mediation effect: study 1: *β* = −0.08, *P* = 0.005; study 2: *β* = −0.05, one-tailed *P* = 0.038; corrected direct effect, study 1: *β* = −0.03, *P* = 0.097; study 2: *β* = −0.03, *P* = 0.12). To obtain a meta-analytical estimate of this mediation analysis, we again pooled data from both studies to establish that our effect was stable across conditions (total effect: *β* = −0.098, *P* = 0.0001; mediation effect: −0.064, *P* = 0.002; corrected direct effect: −0.036, *P* = 0.016). More dogmatic participants also earned less money overall, indicating their lowered information seeking did not entail any strategic benefits (study 1: *β* = −0.24, *P* = 0.008, *R*^2^ = 0.02; study 2: *β* = −0.21, one-tailed *P* = 0.009, *R*^2^ = 0.01; pooled internal meta-analysis: *β* = −0.23, *P* = 0.0003, *R*^2^ = 0.017).

### Trial-by-Trial Modeling of Information Search.

We next sought to develop a more detailed account of how dogmatic individuals’ trial-by-trial information seeking choices were informed by confidence judgments and the cost of information. This model can be expressed as a logistic regression predicting the choice to seek information:

$$P\left(Information\;Seeking\right)=\frac{1}{1+\text{exp}\left(-\left(\beta_{0}+\;\beta_{1}*Confidence+\beta_{2}*Cost\right)\right)}$$

**Fig. 4.**
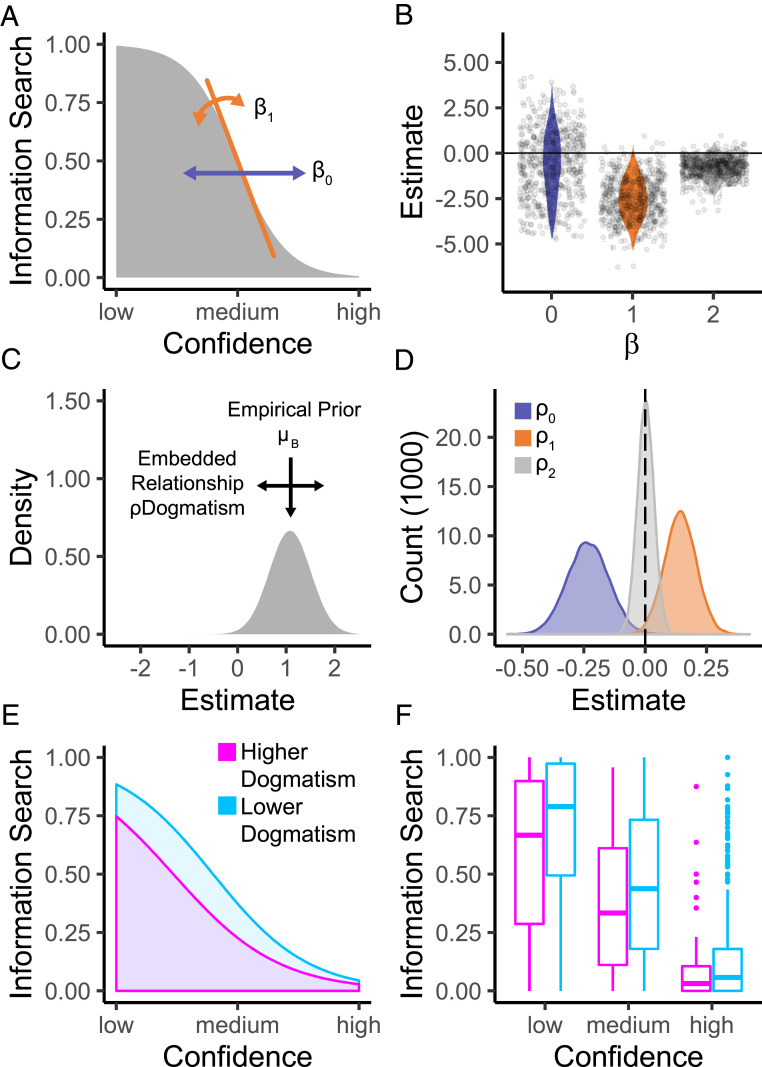
Individual differences in confidence-driven information search as captured by a trial-by-trial model. (*A*) The decision to seek additional information was captured using a model with three parameters: an intercept $\beta_{0}$, a confidence parameter $\beta_{1}$, and a cost parameter $\beta_{2}$ (not depicted here). (*B*) Distribution of individual-level parameters displaying the generally negative influence of higher confidence $\left(\beta_{1}\right)$ and higher cost $\left(\beta_{2}\right)$ on information search. (*C*) We captured dogmatism-related differences in these model parameters through a hierarchical fitting procedure, whereby each parameter’s empirical prior varies as a function of another set of parameters ρ that encode the influence of subjects’ dogmatism scores in a hierarchical estimation scheme. (*D*) Posterior distribution of embedding parameters ρ encoding dogmatism-driven shifts in parameter means. We found a dogmatism-related decrease in the parameter capturing baseline information search $\left(\rho_{0}\right)$ and an increase in the parameter capturing the tuning of information search to participants’ confidence $\left(\rho_{1}\right)$. No effect of dogmatism on the cost parameter was observed $\left(\rho_{2}\right)$. The dotted vertical line represents a null effect. (*E* and *F*) Dogmatic individuals seek out less information than moderates when they are uncertain. To visualize this effect, we compared the 10% most dogmatic participants to the remainder of the sample. We plot (*E*) model predictions and (*F*) actual data (medians with upper/lower quantiles), averaged over both levels of information cost.

The three β’s capture three independent behavioral phenomena (Fig. 4 *A* and *B*): Differences in the model’s intercept, $\beta_{0}$, represent a general shift in willingness to seek out information; $\beta_{1}$ represents how strongly participants’ information-seeking choices are influenced by confidence; and $\beta_{2}$ indicates the influence of information cost on subjects’ willingness to seek out more information.

We were interested in whether any of these parameters were associated with individual differences in dogmatism, i.e., whether dogmatism was linked to a general tendency to seek out less information $\left(\beta_{0}\right)$, a differential influence of confidence on information seeking $\left(\beta_{1}\right)$, or an altered sensitivity to information costs $\left(\beta_{2}\right)$. The relation between each parameter, *β*, and dogmatism was thereby estimated directly within a hierarchical framework, such that each individual’s parameter (indexed by *i*) was a function of a group mean $\left({µ}_{B}\right)$ and their dogmatism score $\left(Dogmatism_{i}\right)$. For instance, the relation between dogmatism and a general tendency to seek out less information can be formalized as follows:

$$\beta_{0,i}={µ}_{B_{0}}\;+\rho_{0}*\;{\mathrm{Dogmatism}}_{i}\;+\;\varepsilon_{B_{0}}$$

Here, $\rho_{0}$ describes the relation between dogmatism and $\beta_{0}$, while $\varepsilon_{B_{0}}$ represents individual variation in this parameter that is not explained by dogmatism. If the credible interval of $\rho_{0}$ does not include zero, this indicates a significant association between dogmatism and $\beta_{0}$ (32). In what follows, we report 95% credible intervals.

We found more dogmatic subjects had lower values of $\beta_{0}$ (Fig. 4*D*; $95\%\;CI_{\rho_{0}}\;=-0.40,\;-0.07$), in accordance with our model-agnostic findings that dogmatic participants show lower information-seeking behavior. While we found no association between dogmatism and cost sensitivity $\left(95\%\;CI_{\rho_{2}}=-0.06,\;0.07\right)$, dogmatism was associated with higher values of the confidence parameter $\beta_{1}\;\left(95\%\;CI_{\rho_{1}}=0.02,\;0.27\right)$. Because $\beta_{1}$ values were generally negative (see Fig. 4*B* for distribution of individual parameter values), this positive shift suggests that the information-seeking decisions of individuals with higher levels of dogmatism were less coupled to fluctuations in subjective confidence than those of individuals with lower levels of dogmatism. In other words, participants with higher levels of dogmatism were less likely to use feelings of confidence or uncertainty to guide their search for more information. Together, this dual shift in both $\beta_{0}$ and $\beta_{1}$ parameters combines to produce marked differences in information search under low confidence (high uncertainty). On these trials, individuals with lower levels of dogmatism were more likely to (adaptively) seek out new information compared to individuals with high levels of dogmatism. In contrast, participants with both higher and lower levels of dogmatism showed similar profiles of information-seeking behavior when they were more confident in their decision (Fig. 4 *E* and *F*).

### Information Search and Other Factor Scores.

Given a long-standing debate over diverging cognitive profiles of liberals and conservatives (33–36), we also investigated the relationship between information search and political orientation. Here, we found that position on the political spectrum (right vs. left) was not predicted by a willingness to seek information (study 1: *β* = −0.07, *P* = 0.19; study 2: *β* = −0.07, *P* = 0.23; *SI Appendix*, Fig. S3). Additionally, there was no consistent association between the extremity of political opinion, as indexed by the absolute value of the political orientation, and information-seeking behavior (study 1: *β* = 0.07, *P* = 0.21; study 2: *β* = −0.13, *P* = 0.02; *SI Appendix*, Fig. S3). Similarly, policy-specific political belief superiority was not related to changes in information seeking (study 1: *β* = 0.03, *P* = 0.62; study 2: *β* = −0.07, *P* = 0.24; *SI Appendix*, Fig. S3).

## Discussion

We show that dogmatic individuals are less likely to seek out additional information before committing to a decision. By foregoing this opportunity, they in turn tend to form less accurate overall judgments. Trial-by-trial modeling revealed that two factors drove dogmatic individuals’ altered information seeking: 1) a shift in the general willingness to seek information and 2) a decreased influence of confidence on information-seeking behavior. Together, these effects gave rise to a distinct pattern: Whereas dogmatism had little effect on information seeking after high confidence decisions, more dogmatic subjects were less likely (relative to moderates) to seek out additional information when they were uncertain about their decision.

A key aspect of our results is that we find this disadvantageous pattern of information seeking in a low-level perceptual decision-making task. This stands in contrast to previous studies on information seeking in the political domain that have relied on questionnaires or experimental tasks with overt political content (27, 37). By capitalizing on the neutral valence and personal irrelevance of simple dot stimuli, we could isolate uncertainty-driven information-seeking behavior from possible confounding effects of motivated reasoning. Observing such an effect in this neutral setting is consistent with a proposal that domain-general cognitive factors contribute to real-world attitudes (38–40). Nevertheless, in most real-world decision-making scenarios, it is likely that both motivational and cognitive (uncertainty-driven) effects contribute to biases in information seeking (6), and it is interesting to consider that the latter may even become magnified in the presence of affective influences.

Our trial-by-trial model fits revealed that while participants generally use internal signals of uncertainty (as assayed by confidence ratings) to guide information search, dogmatic individuals did so to a lesser extent. This points to a general alteration in the way that confidence guides actions, a process usually described as metacognitive control (41). Metacognitive control is hypothesized to not only regulate information search, but also other phenomena in which effort must be weighed against accuracy, such as cognitive offloading (42) or speed–accuracy trade-offs (43). From a theoretical perspective, metacognitive control complements metacognitive monitoring (41), which describes a process that gives rise to, and updates, representations of confidence. However, while metacognitive monitoring has received considerable attention from a neural (44, 45) and individual differences perspective (24, 46), metacognitive control processes remain underinvestigated. Such research might therefore provide fruitful for understanding the drivers of altered information search. Further work is needed also to disentangle how different models of confidence formation [such as postdecisional or second-order architectures (47, 48)] affect both monitoring and control processes, and in turn determine the interplay of confidence and information search.

Dogmatic individuals were less likely to seek information in situations of uncertainty compared to their peers. At a single-trial level, this is consistent with basing a final judgment on less evidence, leading to less accurate judgments overall. Because uncertain decisions are also less likely to be correct, this meant dogmatic individuals were less likely to seek out contradictory evidence when they were wrong—a form of confirmation bias. Over a longer time horizon, and in the absence of external feedback (49), such a self-reinforcing feedback loop might in turn lead dogmatic individuals to think that their initial judgments are already sufficiently optimal and that investing in acquiring more information is unnecessary. A useful extension of our work will be to investigate how dogmatic individuals manage information search in situations that span more than one trial and require iterative learning. In such scenarios, adequately managing the exploration/exploitation trade-off is central to effective learning (5, 8), such that small differences in a tendency toward or against uncertainty-driven information search may summate and lead to skewed representations of reality.

While a psychophysical approach provides us with the precise control required to characterize dogmatic individuals’ information search, our task is necessarily contrived relative to real-world decision problems. It remains unknown whether the types of search behavior observed here are representative of real-world search behavior, for instance on the internet (1). However, we can be cautiously optimistic about the generalizability of the current results, given the domain-general nature of our task and recent observations that real-life behavior adheres to cognitive models of uncertainty-based exploration (50). One difference between our paradigm and real-world decisions is the guaranteed helpfulness of future information. The calculus changes when a first source is trustworthy, but future information might be unreliable. In that case, it might be adaptive to rely more heavily on one’s initial judgment, and refrain from seeking new information even when uncertain.

In sum, we highlight a generic resistance to seek out additional information in more dogmatic individuals, a difference that is most marked when initial decisions are uncertain. This is disconcerting in the current cultural landscape. While the internet has heralded access to a plethora of well-vetted information, fake news remains rife (1, 3). In such cases, the mere availability of correcting information might not be enough to prevent the formation of unsupportable beliefs in dogmatic individuals, because even feelings of uncertainty would not trigger corrective information-seeking behavior. On a systemic level, such results suggest that the veracity of first contact with a news story is therefore critical (51, 52). On an individual level, instilling successful uncertainty-based search may be enabled by the extension of training of metacognitive monitoring (53) to also target metacognitive control. Finally, our research shows that psychophysical paradigms in conjunction with trial-by-trial modeling of behavior provide important tools for identifying mechanisms behind dogmatism, polarization, and their consequences (40).

## Materials and Methods

### Online Recruitment and Sample.

Both studies were conducted online and recruited US adults through the online labor marketplace Amazon Mechanical Turk (54–56). They were approved by the Research Ethics Committee of University College London (#1260-003), and subjects gave informed consent.

In study 1, 370 subjects’ data were analyzed (see *SI Appendix* for exclusion criteria). We based this sample size on previous studies conducted to detect interindividual differences in cognition across the political spectrum (24, 38) and in disorders (46). Subjects were paid a basic payment of $1 and earned a bonus of up to $6 based on their adequate completion of the questionnaires and their performance on the information-seeking task (see *Experimental Design*, *Information-seeking task* for a detailed description of the remuneration). Participants were 50% female (49.7% male, 0.3% “other/would rather not say”) and the mean age was 36.62 y (SD, 11.61; range, 19 to 81 y; *SI Appendix*, Fig. S1*A*). In study 2 (replication), we analyzed data from 364 participants with the same payment scheme as in study 1. An a priori power analysis based on the information-seeking effect size from study 1 determined our sample size in study 2, giving us a power above 80% to detect the association between dogmatism and average information search. The sample consisted of 52% women (47% male, 1% other/“would rather not say”; mean age, 36.55 y; SD, 11.09; range, 18 to 74 y; *SI Appendix*, Fig. S1*A*). Participants in both studies came from a broad range of educational backgrounds, which was comparable to the general US adult population (*SI Appendix*, Fig. S1*B*).

### Factor Analysis.

There is significant debate about the exact structure of political ideology and its relationships to related constructs such as dogmatism (22, 25, 57). As used previously (24), here we administered multiple questionnaires measuring political orientation, identification with the two major US parties, the social and economic conservatism scale (58), as well as a questionnaire assessing specific policy positions and subjects' belief in the superiority of these positions (22). Additionally, right- and left-wing (59, 60) authoritarianism was assessed. Finally, participants also filled out a dogmatism questionnaire (21). We conducted a factor analysis (see *SI Appendix* for a detailed discussion of the results) using the fa() function in the “Psych” R package on all 78 questionnaire items using maximum-likelihood estimation with an oblique rotation (oblimin). This mirrors methodology previously employed to study political beliefs (24) and mental health (46). We determined the number of factors through the Cattell–Nelson–Gorsuch test (61) where a sharp drop in the eigenvalues indicates the point at which there is little benefit to retaining additional factors. To maximize the precision of the factor loading estimates and the factor scores, we pooled the present sample with the one from Rollwage et al. (24) where subjects had completed the same questionnaire battery. This resulted in total sample of 2,135 participants for the factor analysis. We observed qualitatively similar pattern of factor loadings for both the pooled sample of 2,135 participants and the two individual samples.

### Experimental Design.

#### Stimuli.

We used the JavaScript library JsPsych (version 5.0.3) (62) to program the task and hosted the experiment on the online research platform Gorilla (63), which subjects could access through their browser. Two black squares, each 250 pixels in height and width, were presented as discrimination stimuli, with one square positioned left and one square right of center (see Fig. 3 for task overview). Each square consisted of 625 cells, randomly filled with white dots, so that one baseline square always held 313 dots and the other target square contained a greater number determined during a calibration phase (*SI Appendix*). During each dot-discrimination trial, subjects were presented with five such configurations for 150 ms each in order to create the impressions of flickering dots. Within each trial, the location of the individual dots per configuration within one square was random. However, the difference in number of dots between the target and baseline squares remained the same within each trial. The location of the target was pseudorandomized between trials.

#### Task and procedure.

Both studies followed the same protocol and participants spent around 45 min on the experiment, which was divided into three parts. Participants first received information and reported their demographic information. Following this, they then first completed a 120-trial calibration phase to individually determine task difficulty (*SI Appendix*), identical to previous procedures (24). There, participants simply had to indicate which of the two flickering dots contained more dots by a press of the “2” or “6” key (indicating left and right) and received feedback about their correctness through a colored frame around their chosen option. This was followed by the information-seeking task (Fig. 2) in which subjects received no feedback about the correctness. The information-seeking task consisted of four blocks, each containing 25 trials. Participants then went on to fill out the aforementioned questionnaires.

#### Information-seeking task.

Across the 100 trials of the information-seeking task, participants were presented with the stimulus strength determined in the calibration phase (study 1: mean, 73.80%; SD, 6.57%; study 2: mean, 73.67%; SD, 6.50%). As in the calibration phase, participants had to decide whether more dots were in the left or in the right box (the initial decision). Simultaneously, they indicated their confidence in this decision by pressing one of three buttons per side to indicate low, medium, or high confidence (the “1” to “3” or “5” to “7” keys in the number row). Crucially, the information-seeking task allowed participants to choose whether they wanted to see a second, additional display of the stimulus to improve the accuracy of their initial judgment. Subjects were specifically instructed about the helpful nature of this information. If they decided to see the stimulus again, the subjects saw a stronger version of the stimulus (i.e., one with a higher dot difference; *SI Appendix*). If they decided to forego the second stimulus, they were instead presented with two empty black boxes, to prevent them from artificially speeding through the task. Therefore, the only cost associated with the additional information was the deduction of points (5 points or 20 points, depending on the block). Regardless of whether subjects decided to see additional information or not, they then made another judgment (the final decision), indicating both the side they believed contained more dots and their confidence in this final decision (using the same response keys as for the initial decision). Importantly, we only incentivized the accuracy of this final decision: Subjects received 100 points for a correct and 0 points for an incorrect final decision.

Participants’ bonus payment was linked to their performance in the task: They received a $2 bonus for completing the task and an extra 4 cents for every 100 points they had earned on the task (average points-based bonus, study 1: mean, $3.11; SD, $0.34 ; study 2: mean, $3.11; SD, $0.35).

### Statistical Analysis.

#### Task analysis.

We conducted several analyses to ensure participants understood the task and were able to perform it adequately (see *SI Appendix*, Fig. S1 for an overview). Within-participant effects (see *SI Appendix*, Table S1 for an overview) were investigated using trial-by-trial hierarchical mixed effects models computed and analyzed in the “afex” package (64). Specifically, we constructed logistic models with binary outcomes as respective dependent variables and the corresponding predictors as fixed effects (see *SI Appendix*, Table S1 for details). We included per-participant random intercepts and slopes and employed likelihood-ratio tests to obtain *P* values (65). To quantify relationships between subjects’ average information seeking and their final decision accuracy, we set up a general linear model using the lm() function in R. All analyses were performed separately for the two studies.

#### Statistical analysis.

We conducted the following regression analysis using the lm() function in R. All analyses were performed separately for the two studies, and effects were tested two-tailed if not stated otherwise.

1) To investigate the relationship between the factor scores themselves, we constructed polynomial regression models. Specifically, we built these models for each possible factor combination and compared 1) a linear fit, 2) a quadratic fit, and 3) a combined linear and quadratic fit based on their Bayesian information criterion (see *SI Appendix*, Table S2 for an overview).
2) To investigate the relationship between information seeking and the factors observed through our questionnaire, we set up one generalized linear model per factor, explaining the respective variance in this factor score through participants’ average information seeking. Following previous work (24), we controlled for the following covariates: age, gender, education, subjects’ average performance and confidence level on the initial decision, objective stimulus strength (indicated by the logarithm of the dot difference), and performance on the stronger version of the stimulus (as recorded during the calibration phase; see *SI Appendix*). We standardized the continuous outcome and predictor variables to obtain standardized *β* coefficients. For significant variables of interest, we calculated *R*^2^ values by comparing the variance explained by a full model including information seeking relative to a model excluding this predictor.
3) Finally, to check whether dogmatism was associated with a reduction in points earned on our task, we set up the same model used for the information-seeking analysis but replaced the information-seeking predictor with the points earned on the task.

To investigate whether dogmatism was linked to a reduction in final decision accuracy and whether this arose from a lowered propensity to seek out information, we conducted a mediation analysis. This analysis was conducted using the “mediate” package in R (66), which uses a quasi-Bayesian Monte Carlo method based on normal approximation to estimate the significance of the mediation effect (67). We again entered the covariates used for the original information-seeking analysis as control variables into all paths of the mediation analysis. To conduct an internal meta-analysis of behavioral results obtained across the two studies, we pooled the two samples as recommended by Braver et al. (31) and applied the same analysis as detailed above.

#### Trial-by-trial modeling.

To probe the underlying mechanisms contributing to dogmatic individuals’ information search, we set up a trial-by-trial model that investigated the factors impacting an individual’s decision to seek out more information. Specifically, we modeled the information-seeking choices as a function of the confidence level and the current information cost (see main text and *SI Appendix*).

Because classical maximum-likelihood–based methods can frequently provide noisy estimates with so few data points, we employed a hierarchical fitting procedure (68). In such a hierarchical model, individual parameters, $\beta_{i},$ are drawn from a group-level prior distribution. For example, for the first parameters, $\beta_{0,i}$, we can write as follows:

$$\beta_{0,i}∼N\left({µ}_{B_{0}},\;\sigma_{B_{0}}\right)$$

Here, ${µ}_{B_{0}}$ represents the population mean that then informs the estimation of $\beta_{0,i}$, the individual parameters of $\beta_{0}$ for participant *i*, from a population distribution, $N\left({µ}_{B_{0}},\;\sigma_{B_{0}}\right)$. Conventionally, parameters obtained through such an approach can then be correlated with an external measure of differences between individuals. However, this procedure is suboptimal because it assumes no variability in the mean of the population in the initial model fit, possibly distorting or minimizing potential relationships between the parameter and external factors (32). To maintain the advantages of hierarchical fitting while avoiding such pitfalls regarding individual differences, here we employ a procedure recently prescribed by Moutoussis et al. (32). There (Fig. 4*C*), the relationship between the parameters and individual differences is embedded into the estimation of the parameters themselves through the prior, so that:

$$\beta_{0,i}\;∼\;N\left({µ}_{B_{0}}+\;\rho_{0}*\;Dogmatism_{i},\;\;\sigma_{B_{0}}\right)$$

To capture interindividual differences in the parameter, we allow the mean of the population distribution to vary as a function of dogmatism through the embedded parameter $\rho_{0}$. To enable accurate hierarchical estimation, we pooled the samples from both studies and only included subjects that sought out information on at least 5% and at most on 95% of trials. In doing so, we achieved a total sample of 568 subjects. We built the model using the programming language Stan (69), which uses a form of Markov chain Monte Carlo sampling, Hamilton Monte Carlo sampling, to estimate posteriors over parameters.

Further details on model fitting and a discussion of the influence of different parameters on participant’s payoff are presented in *SI Appendix*.

## Supplementary Material

Supplementary File

## Data Availability

Fully anonymized data are available from the corresponding author upon reasonable request. The final code for data analysis has been deposited in a dedicated GitHub repository (https://github.com/metacoglab/SchulzRollwageDolanFleming).
