# Supplementary material for: Dogmatism manifests in lowered information search under uncertainty
Source: Proc Natl Acad Sci U S A. 2020 Nov 19;117(49):31527–34. doi: 10.1073/pnas.2009641117 (PMC7733856; doi:10.1073/pnas.2009641117)
Supplement: Supplementary File [file pnas.2009641117.sapp.pdf]

Supporting Information

Dogmatism manifests in lowered information search under uncertainty

Lion Schulz[a,b,c,1,2], Max Rollwage[a,d,1], Raymond J. Dolan[a,d], and Stephen M. Fleming[a,b,d,2]

[a] *Wellcome Centre for Human Neuroimaging, Institute of Neurology, University College London, London WC1N 3BG, United Kingdom*
[b] *Department of Experimental Psychology, University College London, London WC1H 0AP, United Kingdom*
[c] *Department of Computational Neuroscience, Max Planck Institute for Biological Cybernetics, 72012 Tübingen, Germany*
[d] *Max Planck University College London Centre for Computational Psychiatry and Ageing Research, Institute of Neurology, University College London, London WC1B 5EH, United Kingdom*
[1] *L.S. and M.R. contributed equally to this work.*
[2] *Corresponding authors: LS: lion.schulz@tuebingen.mpg.de, SMF: stephen.fleming@ucl.ac.uk*

www.pnas.org/cgi/doi/10.1073/pnas.2009641117

Detailed sample information

Further sample details. Both studies consisted of participants from a wide range of educational attainments (Fig. S1B) with a distribution similar to the general US population (1). Similar to other studies using Mechanical Turk (2) and our previous study using the same questionnaire (3), our sample skewed towards the liberal side of the political spectrum as observed by answers to a self-report of political orientation from “very liberal”, 0, to “very conservative”, 100 (study 1: mean = 37.38,SD = 29.40; study 2: mean = 38.19, SD = 29.32). However, a subsequent factor analysis allowed us to recover a wide variability in political convictions and related constructs. Participants in Study 2 also completed the short version of the Obsessive-Compulsive Inventory (4) after finishing the main questionnaire battery; these data are not analyzed here.

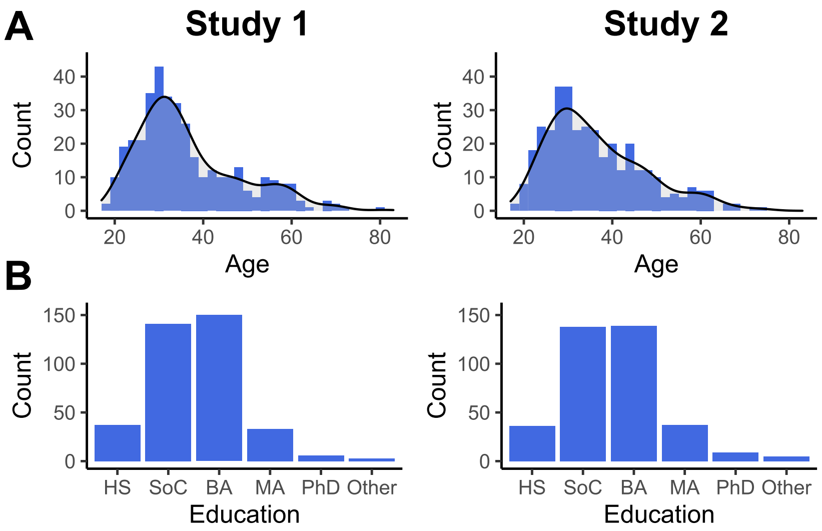

Fig. S1. Demographics for the two studies. Bar plots showing the distribution of **(A)** participant’s ages and **(B)** educational attainments. Abbreviations: HS, High School; SoC, some college; BA, Bachelor’s degree; MA, Master’s degree.

Data quality and exclusion criteria. Mechanical Turk allowed us to gain access to a sample much more diverse than typical college student populations (2). The platform is also known to provide good data quality (5) and internal as well as external validity (6, 7), even with complex tasks (8). Subjects were prevented from multiple participation through a filter option during the job advertisement. We also prevented subjects who had previously taken part in a similar study in our lab from further participation (3).

For study 1, we collected data from 500 participants. Out of this sample, we excluded 130 subjects to obtain a final analyzable sample of 370 participants, an exclusion rate comparable to analogous online studies in our lab (3, 9) and consistent with a recent meta-analysis showing a typical exclusion rate between 3 % and 37 % for web-based experiments (10). Exclusion criteria were defined *a priori* and were based on previous studies from our lab and others (3, 9–11). For study 1, 6 subjects were excluded for failing to correctly respond to at least one of two catch questions that were placed randomly within the questionnaires. Furthermore, we excluded 78 subjects due to their perceptual performance in the initial decision falling outside of an interval between 60 % and 85 % correct indicating non-convergence of the staircase procedure (see below). An additional 34 participants were excluded because they chose the same confidence rating on more than 95 % of trials. We also excluded 12 participants whose data contained more than 5 % of total trials with missing data.

For study 2, we again collected data from 500 participants. Out of this sample, we excluded 136 subjects, leaving us with a final sample of 364 subjects using the same exclusion criteria as for study 1. Specifically, we excluded 9 subjects for failing to correctly respond to the catch questions. A further 85 subjects were excluded due to their perceptual performance falling outside of our predefined range. We additionally excluded 36 subjects because they had chosen the same confidence rating on more than 95 % of trials. Finally, we excluded six participants whose data contained more than 5 % of trials with missing data.

Information-seeking task

Behavioral results. We detail the hierarchical logistical models used to establish whether subjects adequately performed the task in Table S1. The underlying data are presented in Fig. S2.

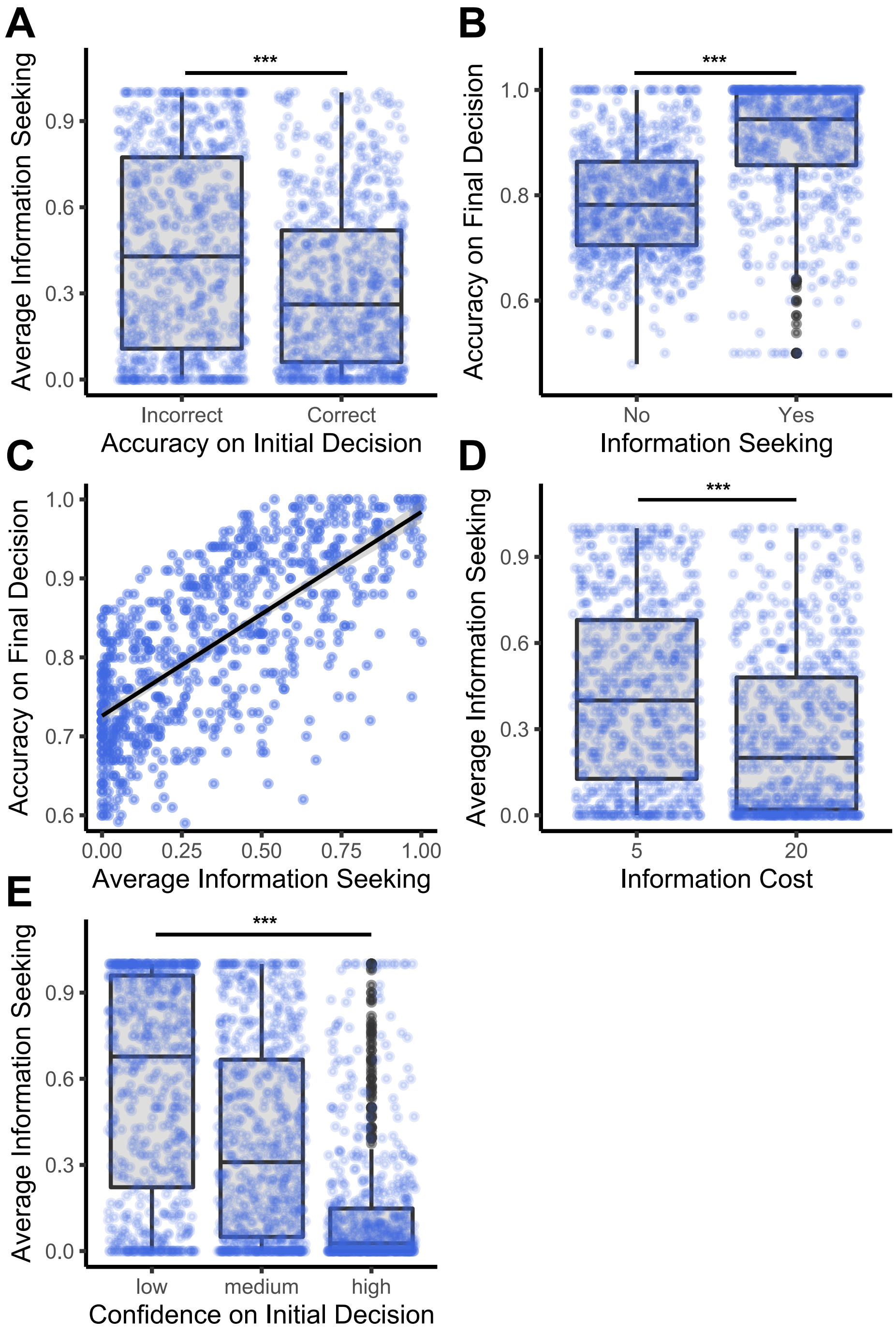

Fig. S2. Behavioral results for information-seeking task. (A) Participants were less likely to seek out information after a correct initial decision. **(B)** On a within-subject level, a participant was more likely to be correct in the final decision after having decided to see the additional information and **(C)** on a between-subjects level participants who used the additional information more often tended to be more accurate in the final decision. **(D)** Higher cost of information lowered participant's willingness to seek out additional information. **(E)** Participants reduced their information seeking as a function of their initial decision confidence. *** p < .001.

Table S1. *Hierarchical logistic regression models (see main text for results).*

Criterion	Predictor	Expected Effect
See-again decision (yes/no)	~ Accuracy on initial decision (incorrect/correct)	negative
Accuracy on final decision (correct/incorrect)	~ See-again decision (yes/no)	positive
See-again decision (yes/no)	~ Information cost (-5/-20 points)	negative
See-again decision (yes/no)	~ Initial decision confidence (low/medium/high)	negative

Calibration phase. Before the main information-seeking task, subjects completed a calibration phase consisting of 120 trials. The purpose of the calibration phase was to approximate an individual stimulus strength, i.e., dot difference between the baseline and target square, for each participant that corresponded to approximately 71 % accuracy in the dot discrimination task phase (actual performance, study 1: mean = 73.80 %, SD = 6.57 %; study 2: mean = 73.67 %, SD = 6.50 %). To achieve this we employed a 2-down-1-up staircase procedure (12) on the logarithm of the dot difference. Each subject completed 70 trials of the staircase. The mean dot difference from the last 25 staircase trials was then set as the constant dot difference for the information-seeking phase of the task.

Two different stimulus strengths were used for the information-seeking phase (one for pre-decision evidence and a stronger level for post-decision evidence, see Fig. 2). Specifically, the pre-decision evidence strength was set to the dot difference obtained from the above described staircase. The post-decision evidence strength was defined as 130 % of the staircased dot-difference in log-space. To probe the subject's performance level at this stronger stimulus strength, we interleaved 50 additional trials in the calibration phase after an initial 20 trials of the regular staircase. The evidence strength of these interleaved trials was set to 130% of the current dot difference of the staircase. Across all participants, this stronger interleaved stimulus strength elicited a mean accuracy of 90.50 % (SD = 7.89 %) in study 1 and 90.07 (SD = 7.21 %) in study 2.

Factor analysis

Results. The first factor tracked political orientation (liberal to conservative). This was indicated by the highest loading item "*Please rate your overall political attitude on the dimension from 'liberal' to 'conservative'"* (factor loading = .91). In addition to tracking general political orientation, this factor also captured more specific policy positions, e.g. about immigration (factor loading = -.71), and parts of the right-wing authoritarianism scale, e.g. "*What our country really needs instead of more 'civil rights' is a good stiff dose of law and order*" (factor loading = .63) and the left-wing authoritarianism scale (e.g.: *"I agree with the basic idea of communism of overthrowing the Establishment – with or without violence- and giving its wealth to the poor"*, factor loading = -.56). Following previous research into the structure of US political orientations, we did not observe a divergence between economic and social conservatism. We note that this conjunction might not hold internationally (13).

A second factor most heavily loaded onto items related to domain-general dogmatism. This is exemplified by the highest loading question *"I am so sure I am right about the important things in life, there is no evidence that could convince me otherwise"* (factor loading = .75), or the second highest loading item, "*There are no discoveries or facts that could possibly make me change my mind about the things that matter most in life*" (factor loading = .71). Additionally, this factor received mild contributions from questions about policy issues such as the influence of religion on laws (factor loading = -.44), consistent with a close link between dogmatism and religiosity (14).

A final, third factor was related to policy-specific belief superiority. The top nine highest loading items originated from the belief superiority questionnaire with factor loadings between .52 and .61. The highest loading item for this factor was: "*In your view, how much more correct are your beliefs about the government's role in helping people in need than other people's beliefs about this issue?"* (factor loading = .61).

Based on these loadings, we labelled the first factor "*Political Orientation*", the second factor "*Dogmatism*" and the final factor "*Political Belief Superiority*". The identification and labelling of the three factors reflect a combination of data- and theory-driven approaches, but we recognize that this labelling is inherently subjective and that alternate names are possible.

Table S2. *BIC scores for models of relationships between factor scores (see main text and methods for further details).*

Criterion	Predictor	Study 1 BICs	Study 2 BICs
Dogmatism	~ Political Orientation	1036.67	998.29
	~ (Political Orientation)^2	988.03	963.64
	~ Political Orientation + (Political Orientation)^2	983.35	944.65
Political Belief Superiority (PBS)	~ Political Orientation	1054.94	1033.40
	~ (Political Orientation)^2	1008.83	1025.77
	~ Political Orientation + (Political Orientation)^2	970.42	993.46
Dogmatism	~ PBS	1040.74	1042.19
	~ (PBS)^2	1066.60	1047.27
	~ PBS + (PBS)^2	1045.30	1047.76

Discussion of dogmatism and the general factor structure in relation to previous investigations We note that the factor structure obtained using data from the current study (N = 734), as well as pooling data with our previously published study (24, N = 2,135), is qualitatively different from that obtained in our previous study (3), despite using the same set of questionnaires and factor analytic approach. Two key changes surfaced in our newer analyses: (1) Political orientation and authoritarianism were unified in one factor, and (2) dogmatism and political belief superiority were separated into two factors. Potential reasons for these differences are discussed below.

First, our political orientation factor includes items from the authoritarianism scales, unlike in Rollwage et al. where the left- and right-wing authoritarianism questionnaires loaded most heavily on a separate factor that was labelled “authoritarianism”. However, it is noteworthy that the political orientation and authoritarianism factors were correlated, hinting at an underlying relationship, even in Rollwage et al’s (2018) data. One possible explanation behind this change in factor structure is a change in the structure of US political attitudes over time. Policy positions and partisan identifications might have unified increasingly into one continuum that now encompasses left- and right-wing authoritarianism. This is congruent with a trend in U.S. politics that has seen an increasing division into two clearly defined camps, each endorsing more extreme beliefs over time (15, 16).

Second, a factor termed “dogmatic intolerance” by Rollwage et al (2018) encompassed items from two factors that were separately identified in the present study, a domain-general “dogmatism”

factor and a policy-focused “political belief superiority” factor. While these two constructs might initially seem highly related, a separation along these lines is consistent with theoretical perspectives on dogmatism. Specifically, whereas dogmatism describes a general certainty in one’s attitudes (14, 17), political belief superiority involves a judgement of other people’s specific policy opinions (18, 19) and varies between issues even within the same individual (20).

Relationships between dogmatism and age

As shown in Fig. 3A, we observed an effect of age on dogmatism in study 2 ($\beta = .25$, $p < 10^{-5}$) but not study 1 ($\beta = 0.026$, $p = 0.628$). There was no significant difference in the age distribution (Kolmogorov-Smirnov test: $D = 0.04$, $p = 0.90$) that might explain this discrepancy in age effect between both samples. Consequently, we conducted additional analyses to ensure that the link between dogmatism and information seeking in study 2 was not confounded by age.

First, in both samples there was no significant association between age and information seeking (study 1: $\beta = .026$, $p = .617$; study 2: $\beta = .099$, $p = 0.060$). Second, we tested whether there was an age × information seeking interaction effect that could impact our conclusions regarding the association between information seeking and dogmatism. This interaction effect was not significant in both samples (study 1: $\beta = .002$, $p = .960$; study 2: $\beta = .075$, $p = .125$). Lowered information seeking remained a predictor of dogmatism (study 1: $\beta = -.154$, $p = .006$; study 2: $\beta = -.107$, one-tailed $p = 0.027$) when including this interaction. Together these analyses indicate that the differential association between age and dogmatism between the two samples does not impact our main conclusion regarding reduced information seeking in more dogmatic participants.

Trial-by-trial modeling

Model fitting. We used Hamilton Monte Carlo sampling implemented in STAN (21) to approximate draws from the posterior distribution of parameters. Due to the relatively low number of trials available per participant, a hierarchical fitting procedure was employed where relationships between parameters and dogmatism were embedded into the fitting itself (see Methods). We used 4 chains, each with 10000 iterations and 5000 warm-up, a conservative approach compared to STAN’s default

setting (2000 iterations, 1000 warm-up). R-hats for the parameters were all ≈ 1 indicating good convergence. The following priors were employed for model fitting:

Group-level parameters B_j (same for B_0, B_1, B_2)

$$\mu_{B_j} \sim \mathcal{N}(0\,,10)$$

$$\sigma_{B_j} \sim uniform(0\,,10)$$

Individual level-parameters, β_0, β_1, β_2 for each participant i

$$\beta_{0,i} \sim \mathcal{N}\left(\mu_{B_0} + \rho_0 * Dogmatism_i\,,\sigma_{B_0}\right)$$

$$\beta_{1,i} \sim \mathcal{N}\left(\mu_{B_1} + \rho_1 * Dogmatism_i\,,\sigma_{B_1}\right)$$

$$\beta_{2,i} \sim \mathcal{N}\left(\mu_{B_2} + \rho_2 * Dogmatism_i\,,\sigma_{B_2}\right)$$

Model for each participant i and each trial t

$$InformationSearch_{i,t} \sim Bernoulli\_logit(\beta_{0,i} + \beta_{1,i} * Confidence_{i,t} + \beta_{2,i} * Cost_{i,t})$$

Model plotting. To visualize the impact of dogmatism on uncertainty-guided information search (a posterior predictive check, Fig. 4B), we extracted the average β_0 and β_1 of the participants with the 10% highest dogmatism factor scores compared to the remaining participants. We then plotted the two choice probability functions resulting from models simulated with the parameter values of these two groups:

$$P(Information\ Seeking) = \frac{1}{1 + \exp\left(-(\beta_0 + \beta_1 * Confidence)\right)}$$

For better visualization in two dimensions and because there was no dogmatism-driven shift in β_2, we removed the cost factor. (Cost was coded as -1 for the -5 points blocks and as 1 for the –20 points blocks to visualize information search for an "average" level of cost).

Impact of model parameters on task performance. To investigate whether alterations in the model parameters had an impact on participants' earnings, we constructed a multiple regression analysis predicting earnings from β-parameters. We used the lm() function in R and controlled for accuracy on the initial decision. All variables were standardized, and analysis conducted for the pooled sample. We found a positive influence of β_0 on the maximum points earned ($\beta = .21$, $p < 10^{-17}$), again showing

that participants with a lower baseline tendency for information search earned less money on our task. Similarly, there was a negative influence of β_1 on the points subjects earned in the task (β = -.14, $p < 10^{-9}$). This demonstrates that a lesser tuning of a participant's information search to changes in subjective uncertainty had a negative influence on earnings. Finally, we found a small effect of β_2 on the number of points earned (β = -.05, p = .04), highlighting that participants who were more sensitive to changes in the cost of the second piece of information (i.e., had a lower β_2) also earned more points.

GLM Results

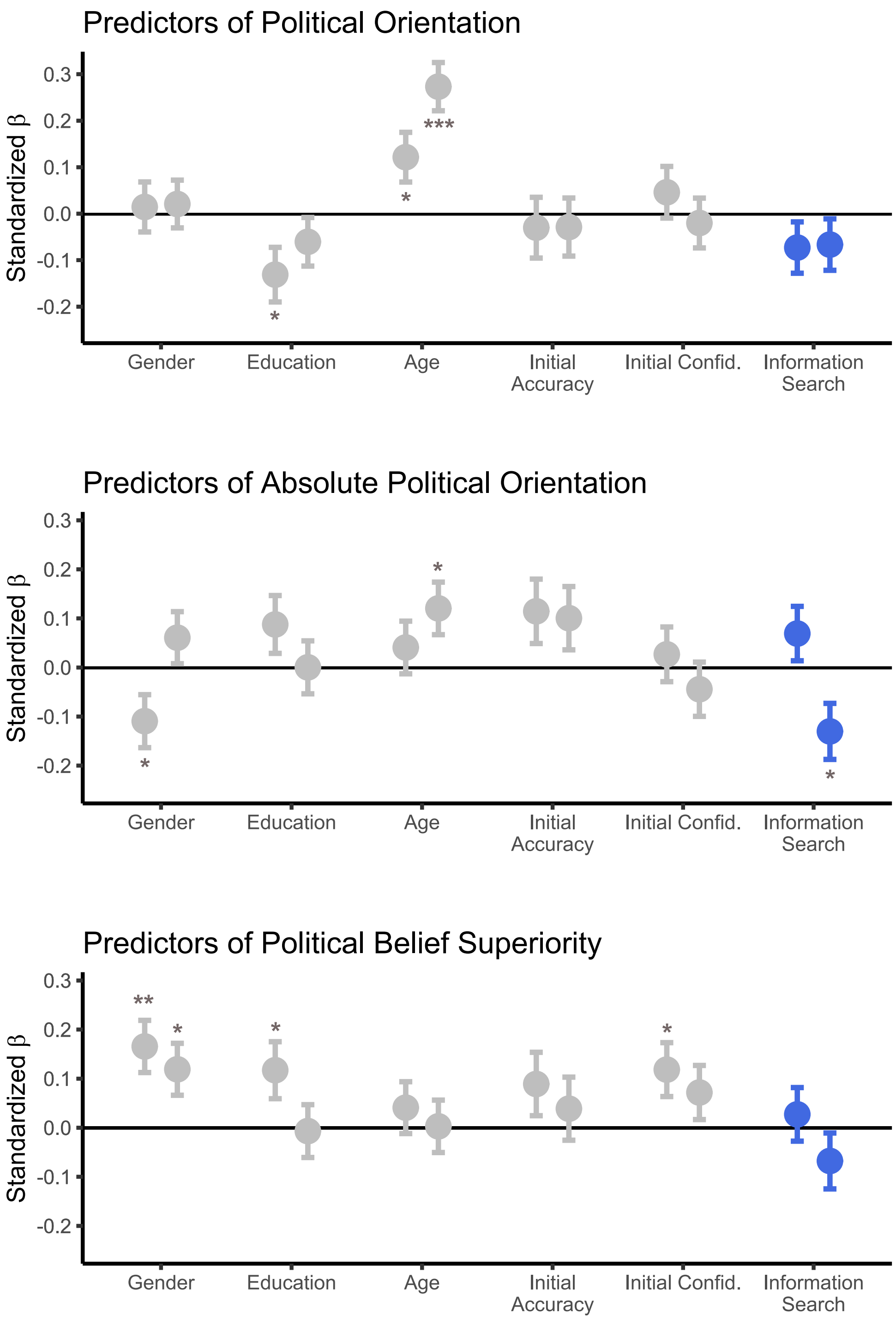

Fig. S3. Results of a GLM predicting political orientation, absolute political orientation and political belief superiority. Political orientation, absolute political orientation, and political belief superiority were not consistently predicted by alterations in information seeking. We present standardized beta coefficients ± standard error of predictors for study 1 (left markers, N = 370) and study 2 (right markers, N = 364). *p<.05, **p<.01, ***p< .001.

References for SI

1. C. L. Ryan, K. Bauman, "Educational Attainment in the United States: 2015" (2016).
2. C. Huff, D. Tingley, "Who are these people?" Evaluating the demographic characteristics and political preferences of MTurk survey respondents. *Res. Polit.* **2** (2015).
3. M. Rollwage, *et al.*, Metacognitive Failure as a Feature of Those Holding Radical Beliefs. *Curr. Biol.* **28**, 4014-4021.e8 (2018).
4. E. B. Foa, *et al.*, The Obsessive-Compulsive Inventory: Development and validation of a short version. *Psychol. Assess.* **14**, 485–496 (2002).
5. M. Buhrmester, T. Kwang, S. D. Gosling, Amazon's Mechanical Turk: A new source of inexpensive, yet high-quality data? *Perspect. Psychol. Sci.* **6**, 3–5 (2011).
6. D. G. Rand, The promise of Mechanical Turk: How online labor markets can help theorists run behavioral experiments. *J. Theor. Biol.* **299**, 172–179 (2012).
7. J. J. Horton, D. G. Rand, R. J. Zeckhauser, The online laboratory: conducting experiments in a real labor market. *Exp. Econ.* **14**, 399–425 (2011).
8. M. J. C. Crump, J. V. McDonnell, T. M. Gureckis, Evaluating Amazon's Mechanical Turk as a Tool for Experimental Behavioral Research. *PLoS One* **8**, e57410 (2013).
9. M. Rouault, T. Seow, C. M. Gillan, S. M. Fleming, Psychiatric Symptom Dimensions Are Associated With Dissociable Shifts in Metacognition but Not Task Performance. *Biol. Psychiatry* **84**, 443–451 (2018).
10. J. Chandler, P. Mueller, G. Paolacci, Nonnaïveté among Amazon Mechanical Turk workers: Consequences and solutions for behavioral researchers. *Behav. Res. Methods* **46**, 112–130 (2014).
11. D. M. Oppenheimer, T. Meyvis, N. Davidenko, Instructional manipulation checks: Detecting satisficing to increase statistical power. *J. Exp. Soc. Psychol.* **45**, 867–872 (2009).
12. M. A. García-Pérez, Forced-choice staircases with fixed step sizes: Asymptotic and small-sample properties. *Vision Res.* **38**, 1861–1881 (1998).
13. A. Malka, Y. Lelkes, C. J. Soto, Are Cultural and Economic Conservatism Positively Correlated ? A Large-Scale Cross-National Test. *Br. J. Polit. Sci.* **49**, 1045–1069 (2019).
14. B. Altemeyer, Dogmatic behavior among students: testing a new measure of dogmatism. *J. Soc. Psychol.* **142**, 713–721 (2002).
15. A. Kohut, C. Doherty, M. Dimock, S. Keeter, "Partisan Polarization Surges in Bush, Obama Years" (2012).
16. C. Doherty, J. Kiley, B. Johnson, "The Partisan Divide on Political Values Grows Even Wider" (2017).
17. J. Duckit, "Authoritarianism and dogmatism" in *Handbook of Individual Differences in Social Behavior*, M. R. Leary, R. H. Hoyle, Eds. (The Guilford Press, 2009), pp. 298–317.
18. K. Toner, M. R. Leary, M. W. Asher, K. P. Jongman-Sereno, Feeling Superior Is a Bipartisan Issue: Extremity (Not Direction) of Political Views Predicts Perceived Belief Superiority. *Psychol. Sci.* **24**, 2454–2462 (2013).
19. R. E. Petty, P. Briñol, Z. L. Tormala, D. T. Wegener, "The role of metacognition in social judgement" in *Social Psychology: Handbook of Basic Principles*, A. W. Kruglanski, E. T. Higgins, Eds. (The Guilford Press, 2007), pp. 254–284.
20. D. Schkade, C. R. Sunstein, R. Hastie, When Deliberation Produces Extremism. *Crit. Rev.* **22**, 227–252 (2010).
21. B. Carpenter, *et al.*, Stan: A Probabilistic Programming Language. *J. Stat. Softw.* **76**, 1–32 (2017).
